# Exploring computer-aided health decision-making on cervical cancer interventions through deliberative interviews in Ethiopia

**DOI:** 10.1038/s41746-023-00808-9

**Published:** 2023-04-17

**Authors:** Frithjof Sy, Astrid Berner-Rodoreda, Takelech Asnake, Misrak Getnet, Wondwossen Amogne, Hermann Bussmann, Helen Abera, Till Bärnighausen, Andreas Deckert

**Affiliations:** 1 Heidelberg Institute of Global Health, University of Heidelberg, Heidelberg, Germany; 2 Federal Ministry of Health, Addis Ababa, Ethiopia; 3 Ethiopian Public Health Institute, Addis Ababa, Ethiopia; 4 Infectious Disease Department (TASH) Addis Ababa University, Addis Ababa, Ethiopia; 5 Institute of Pathology, University of Heidelberg, Heidelberg, Germany; 6 Harvard Center for Population & Development Studies, Harvard University, Cambridge, MA USA; 7 Africa Health Research Institute, KwaZulu-Natal, South Africa

**Keywords:** Decision making, Viral infection, Cervical cancer, Cancer prevention, Computational science

## Abstract

Cervical cancer is a significant disease burden in Ethiopia. Mathematical models and computer simulations on disease dynamics can support effective resource allocation. The objectives of this work are (i) to explore the perspectives of health decision-makers on computer-aided predictions supporting cervical cancer interventions, (ii) to identify their information needs from these predictions, and (iii) their willingness to apply the results in their work. We conducted deliberative interviews with 15 health decision-makers and advisors in Ethiopia in autumn 2019. We analyze the data using a five steps framework approach drawing on thematic analysis and find that Ethiopian health decision-makers are willing to use computer-aided predictions in their decisions. Data on HPV prevalence and the cervical cancer burden are scarce but valued highly and decision-makers are particularly interested in the identification of local HPV hotspots. Data-driven mathematical models and computer simulations may increasingly influence health decision-making in Ethiopia.

## Introduction

Among cancers affecting women, cervical cancer (CC) ranks fourth in the world’s list of the deadliest cancers for women affecting 604,000 individuals and claiming 342,000 lives globally in 2020^1^. With the help of targeted prevention programs, high-income countries were able to drastically decrease CC deaths in recent decades^2^. In contrast to the decline of the CC burden in the western world, CC remains a considerable problem in resource-constraint countries due to a lack of proper infrastructure for screening and vaccination^1^. This problem is particularly pronounced on the African continent where many countries face a high age-standardized rate (ASR) of CC incidence and mortality^1^. As of 2020, Ethiopia has an ASR for CC incidence of 21.5 per 100,000 and 16.0 per 100,000 CC mortality. With 7445 new cases and 5338 deaths annually, CC ranks second after breast cancer among women’s most common cancers in Ethiopia^3^.

Invasive CC is associated with persistence of high-risk human papillomavirus (HPV) strains^4,5^ therefore screening and vaccination against HPV are of utmost importance. Like many African countries, Ethiopia struggles with scarce resources for health care. In 2019, the total health expenditure was about 3% of the Ethiopian gross domestic product^6^, highlighting the need for prudent resource allocation in order to achieve better societal health outcomes.

Mathematical models have already aided in the appropriate allocation of scarce resources in a variety of scenarios, including low- and middle-income countries (LMICs), as well as high-income countries around the world. The WHO guideline for the screening and treatment of cervical pre-cancer lesions for cervical cancer prevention was developed using mathematical models, such as the Policy1-Cervix platform. The models employed also contained Tanzanian-specific work^7,8^. As the scarcity or absence of data makes decision-making difficult, Hontelez and colleagues discussed the case of HIV program integration. They propose to draw on well-established design and implementation methodologies, such as systems-thinking processes, accompanied by deliberation and iterative improvement of local service delivery strategies^9^.

The prediction of the spread of infectious diseases such as HIV infections, COVID-19, and also HPV infections can be of help for health decision-making^10–13^. Modeling HPV infections and cost-effectiveness of HPV and CC interventions have been conducted for high- income countries like Switzerland and LMICs like Kenya or Peru^13,14^. Goldie and colleagues investigated the cost-effectiveness of CC screening interventions in five low-income countries using a state transition model^14,15^. Riesen and colleagues modelled effects of regional uptake of HPV vaccination in Switzerland using ordinary differential equations (ODEs)^13^. Similarly, Tracy and colleagues modelled the impact of a single vaccination campaign of all at-risk women in Mali, focusing on the serotypes HPV 16 and 18, based on ODEs^16^. HPV transmission models as used by Riesen and Tracy utilize dynamic equations which vary over time. The starting point for understanding these models are simple susceptible – vaccinated – infected – recover (SVIR) – models consisting of four compartments that each represent a population state. Individuals can change e.g. from a susceptible to an infected individual, after recovery the individual moves to the recover-compartment and becomes part of the healthy population once more. Hughes and colleagues described an even easier SIR model in 2002^17^ where, to adequately model HPV transmission, expansions of such simple models are needed to avoid systematically biasing results by omitting crucial dynamics. Sexual mixing can be accounted for with a mixing matrix, which considers sexual activity groups, sub-populations, and sexes^13^. Examining sexual networks may be of interest^18^.

Furthermore, considering CC screening algorithms or different genotypes, particularly relevant for screening with HPV DNA-testing, can be advantageous^19,20^. Liu and colleagues use a compartment model of HIV and HPV in Kenya to predict CC incidence and HIV prevalence^19^. They adapted their model from Tan and colleagues, who focused on the effectiveness of the HPV vaccination for South African women living with and without HIV^21^. Van Schalkwyk and colleagues additionally considered a CC screening algorithm, concluding with a significant future effect on CC reduction with new screening technologies^20^. Furthermore, Drolet and colleagues studied cost-effectiveness for vaccination scenarios, including for Nigeria and Uganda^22^.

To our knowledge, similar models have not been applied yet in the Ethiopian context and literature on the needs of African health decision-makers, their views and perspectives in the light of mathematical modeling and computer simulations is scarce. Since policymakers may eventually work with these models and predictions, their opinions are of utmost importance.

There is some evidence regarding the perception of health policymakers on electronic health (eHealth) from Ghana focusing on mobile health (mHealth) interventions where policymakers are inclined to apply mHealth strategies in the future^23^. A qualitative study to explore administrators’ and health service providers’ perceptions on functions of district health systems in Ethiopia identified 11 functions of district health-care systems such as the capacity of health centers and health professionals to provide health care and to facilitate access for the provision of health care^24^.

In terms of interview styles, Brinkmann distinguishes “doxastic” and “epistemic”-type interviews^25^. In doxastic interviewing, the interviewer aims at understanding the interviewee’s perspectives by removing his or her attitudes, perceptions and knowledge from the interview;^26,27^ in epistemic interviewing, the interviewer and interviewee exchange ideas and co-construct knowledge^25^. Epistemic interviewing can be traced back to Socrates, who challenged his dialogue partner by highlighting inconsistencies in the argumentation which he felt could lead to possible new insights^25,27^. Berner-Rodoreda and colleagues illustrated the spectrum of interview styles ranging from doxastic to epistemic and introduced a new epistemic interview style - the deliberative interview - in which interviewer and interview partner interrogate each other in a quest for co-constructing knowledge. The interview style resembles a theme-based conversation rather than a “traditional” interview^27^.

Using deliberative interviews^27^, we sought to co-create knowledge with interview participants, looking at different facets of mathematical model predictions in improving decision-making on health interventions. Most health-decision makers either consider using predictive models in their future work or are already using them and consider high-quality data essential for effective decision-making. While the available data is scarce, local data sources such as the DHIS2 and DHS are valued highly. We build knowledge regarding the importance of HPV hotspots and the cost-effectiveness of possible cervical cancer interventions and show that data-driven predictive models tailored to the Ethiopian context could have a growing impact on future health policy in Ethiopia.

## Results

### Sociographic data

The 15 health decision-makers interviewed were aged between 29 and 60 years. Most participants were below the age of 40 years. Respondents consisted of 14 male and one female health decision-makers. All held university degrees: Bachelor (*n* = 1), Master (*n* = 6), MD (*n* = 1), MD and Master (*n* = 4), PhD (*n* = 3). They were working for the Federal Ministry of Health (FMOH) (*n* = 5), the Ethiopian Public Health Institute (EPHI) (*n* = 3) and other Ethiopian institutions like the Ethiopian Biotechnology Institute (EBTI) (*n* = 1), Black Lions hospital (*n* = 1), St. Paul’s hospital (*n* = 3), Family Guidance Association of Ethiopia (FGAE) (*n* = 1); one was a public health officer (*n* = 1). Participants engaged in high-level and low-level decision-making, from FMOH to field workers (*n* = 2). An overview of the sociodemographic data is provided in Table 1.

**Table 1:** Sociodemographic data of participants (Abbreviations: MD Medical doctor, PhD = Doctor of Philosophy).

Parameters	Number (*n* = 15)	Percentage in %
**Age**		
29–40	9	60
41–50	5	33
51–60	1	7
**Gender**		
Male	14	93
Female	1	7
**Seniority (prof. experience in healthcare in years)**		
5–10	4	27
10–15	6	40
15–20	0	0
20–25	5	33
**Education**		
Bachelor	1	7
Master	6	40
MD	1	7
MD and Master	4	26
PhD	3	20
**Level of decision-making**		
Nation-wide	5	33
Local	10	67

The following six sub-themes were identified as important considerations for participants in the context of achieving greater data reliability and dealing with resource constraints: data resources; HPV and CC burden in Ethiopia, HPV screening and vaccination, information for decision-making, significance of mathematical modeling and computer simulations and outlook and recommendations of decision-makers. The themes will be presented in this order.

### Data resources

The theme ‘data resources’ covers the preferred information resources of our participants. Health decision-makers drew on national and international data sources for their work and every decision-maker we talked to spoke of data-scarcity (Box 1, excerpt 1).

In general, decision-makers used international data from the World Health Organization (WHO), particularly from the GLOBOCAN database kept by the International Agency for Research on Cancer (IARC) for planning and writing proposals. Additionally, they said that they rely on international publications from the American College of Obstetricians and Gynecologists (ACOG) and International Gynecologic Cancer Society (IGCS), as well as international conferences on CC and human papillomavirus (HPV) research. One-third of the health-decision makers expressed doubts about the international data sources (Box 1, excerpt 2).

Local data sources were considered more useful by almost every participant in our study. A frequently named source was the District Health Information System 2 (DHIS2), which gathers local data and is run by the Federal Ministry of Health (FMOH) (Box 1, excerpt 3). The Demographic & Health Survey (DHS) implemented by the Central Statistical Agency (CSA) of Ethiopia in collaboration with the FMOH and Ethiopian Public Health Institute (EPHI) is a survey of representative samples on the national and regional level in Ethiopia^28^. The DHS was frequently mentioned by health decision-makers (Box 1, excerpt 4) and other local sources of HPV and CC data specified were a cancer registry in Addis Ababa as an individual effort of local researchers. Moreover, interview participants also highlighted that there are useful local studies from hospitals identifying genotype distribution of HPV infections and cervical cytology abnormalities like Ali and colleagues;^29^ Addis Ababa University, school of public health; community-based surveys and HPV prevalence studies conducted by students; the HPV consortium, which is a collaboration of different public institutions of Ethiopia situated at the Ethiopian Biotechnology Institute (EBTI) in Addis Ababa.

Box 1 Data resources **Excerpts from interviews:**

1. **IP:** We don’t really have comprehensive data for Ethiopia, but we have some bits and pieces of data from different areas. (MED, advisor and decision-maker)
2. **I:** What do you think about the data from WHO, the international agency for research on cancer and the GLOBOCAN data? Would you consider this?
  **IP:** …as a country, if we don’t have enough data of our own. I wonder where WHO gets its data.
  **I:** I wonder, too. Exactly.
  **IP:** You know it’s really hard to tell, if this is really accurate or not, because first of all we don’t even have women that get screened for um.. HPV or [get a] Pap-smear for cervical cancer, like I said. (PHS, advisor and decision-maker)
3. **IP:** So we have, um… collected data in two ways. We have the DHI system, district health information system version 2. It also results that HPV is one of the indicators, HPV vaccination, it comes through that one. The second one is we just collect it from the immunization focal points directly. HPV is conducted two times per year, because two doses for a girl. So we have direct[ly] collected the information from the subnational level. (MOH, decision-maker)
4. **IP:** in [the] Ethiopian setup we are mostly using data from the Ethiopian Public Health Institute (EPHI)
  **I:** ok!
  **IP:** EPHI, um… every month they publish on health issues, different health issues [in] the DHS, Ethiopian demographic health survey. (EPHI, advisor and health decision-maker)

### HPV and cervical cancer burden in Ethiopia

With an estimated 6300 new cases in 2018, health decision-makers regarded CC as the second most frequent malignancy among women in Ethiopia. Economic and gender vulnerabilities were mentioned by interviewees as barriers to utilizing healthcare services and spoke of the issue of patients presenting themselves in a very late stage of the disease to their healthcare providers. According to participants, reasons for seeking healthcare services late might be the fear of not being able to pay for the diagnosis or treatment, being unaware that the service is free or being denied permission by the husband. As one of our participants put it (Supplementary Table 1, excerpt 1):

These barriers towards a quick diagnostic might lead to undetected CC cases and deaths. which are not reflected in the official data - “*because there are people dying in their home…”*. Undiagnosed affected women were thus unable to get help.

The HPV burden in Ethiopia and its geographic focus remained unclear in interviewee’s descriptions. Interviewees hypothesized that urban areas were more likely to be affected than rural areas due to the spread of sexually transmitted diseases (Supplementary Table 1, excerpt 2).

Furthermore, HPV prevalence goes hand in hand with HIV prevalence, which was perceived to be higher in the cities (Supplementary Table 1, excerpt 3).

Ethiopian cross-border traffic was seen as another aggravating factor (Supplementary Table 1, excerpt 4). It was local health decision-makers, mainly male field workers, who provided this information.

### HPV screening and vaccination

All participants found HPV screening and vaccination to be a priority and depending on their expertise, decision-makers were more inclined either towards screening or vaccination as a priority. In regards to vaccination, prevalent genotypes of HPV were considered crucial. The general approach relies on genotypes 16 and 18 as the most predominant types and all decision-makers underscored the importance of HPV-16, but there were doubts upon HPV-18. This is because even though HPV-18 is considered a critical trigger of CC in Ethiopia, health decision-makers had their doubts regarding ranking it second after HPV-16. Interviewees with higher professional positions casted doubts upon HPV-18 because they felt, based on isolated research, there might be genotypes that are more common than HPV-18 (Supplementary Table 2, excerpts 1-2).

The health decision-maker and interviewer then deliberated the significance of HPV type 16 and type 18 for the Ethiopian society and their relevance for incorporating particular kinds into a mathematical model in the following conversation. It appeared that the interview partner changed their mind in this deliberation, as the two interview partners agreed on the necessity of further studies to verify the relative importance of genotype HPV 18. Both parties agreed on focusing on the most prevalent types for integration into a mathematical model (Supplementary Table 2, excerpt 3).

The sharing of the interviewer’s personal experience in a local Ethiopian hospital revealed the interviewee’s position on screening and prevention. The interviewer explained the encounter with a patient suffering from advanced CC without hope for a cure (Supplementary Table 2, excerpt 4).

A joint takeaway for both interview partners was the affirmation of the relative importance of screening and prevention programs on CC.

Furthermore, due to the conversational nature of the interview, a range of religious and cultural variables seemed to present as impacting whether or not a young Ethiopian girl is vaccinated against HPV (Supplementary Table 2, excerpt 5).

As the interview partners stated scarce resources are a concern not only in research but nearly everywhere across the healthcare system. Interviewees perceived a general lack of health professionals (Supplementary Table 2, excerpt 6), and treatment and screening for CC was depicted as limited to big cities leaving the rural areas even more resource-deprived. Long waiting lists for surgery and radiotherapy were discussed with participants describing that Ethiopia currently has only one radiotherapy facility for advanced CC treatment. Furthermore, the total health expenditure in the country was seen as low and particularly low for cancer prevention and treatment. Moreover, they noted that many health initiatives, such as CC preventive programs, are donor-dependent and that the scarcity of resources was regularly leading to ethical dilemmas (Supplementary Table 2, excerpt 7).

### Information for decision-making

To make an informed decision about HPV interventions and CC prevention, health decision-makers discussed the necessity for specific types of information. It emerged that the specific burden of CC, as well as HPV infections, is crucial for health professionals to know on a national and regional level. According to interviewees, researchers have to identify the seroprevalence of HPV on a national level and in local populations and that the information about HPV was required to be as detailed as possible (Box 2, excerpt 1).

Decision-makers would like to have exact information about herd immunity, as well as HPV hot spots which were defined as a town or region with a high prevalence of HPV. Furthermore, they expressed the need for information on the potential risk factors associated with HPV.

The availability of resources, as well as the identification of resource-poor areas, were deemed essential by decision-makers. Above all, it emerged from interviews that health decision-makers were interested in the cost and sustainability of intervention and if the intervention was cost-effective and would have a continuous positive effect on the population (Box 2, excerpt 2). Vaccination thresholds of herd immunity were also of interest to all participants but one. One decision-maker proposed that there should be a vaccination for every girl in the country.

Participants said that the factors that determine the uptake of vaccination or acceptance of health interventions concerning HPV and CC were of importance because they were also worried about who is visiting the health facilities and who is normally interested in receiving assistance versus who is not.

Participants asked that information presented to stakeholders using the predictions be of good quality and backed by reliable data (Box 2, excerpt 3). Results should be displayed clearly and understandably.

Box 2 Information for decision-making **Excerpts from interviews:**

1. **I:** The reproductive number of a virus, in this case HPV. We might be able, for a special population, maybe for a village, to calculate this reproductive number.
  **IP:** I understand.
  **I:** It might also be possible to identify hot spots like HPV hot spots, local hot spots. What do you think about this? Would that be a useful information to know in order to make a decision, or is it not as relevant to you?
  **IP:** Yeah, definitely. It’s very relevant information… That’s very important. Even in order to come up with herd immunity, we need to really identify those areas, specifically. We need to have really important information about HPV currently in different areas, in different groups, in different living types, standards… (EPHI, advisor and health decision-maker)
2. **IP:** …you need to know, the cost of that intervention has to be also considered, and the other thing is, do we have any plan to make it sustainable. (MED, advisor and health decision-maker)
3. **IP:** So, I would suggest that you have to be very critical with your input and the information you are going to put into your equation, because there the equation gives you whatever you put into it. So, sometimes you give erroneous information into your equation, and then you expect the right answer. (MOH, health decision-maker)

### Significance of mathematical modeling and computer simulations

Knowledge of mathematical modeling and computer simulations differed among participants with some being experts in statistical modeling or having at least some experience while others did not have any experience. The majority of health decision-makers found mathematical models and computer simulations useful for their decision-making and would use the information generated in their future work or were using it already. They considered information from these models useful as long as the quality of input data was good (Supplementary Table 3, excerpt 1), and the model had been peer-reviewed. Younger participants argued more enthusiastically in favor of theoretical predictions with computer simulations. Seven of nine participants aged 29–40, and one of six participants aged 41–60, raised fewer concerns towards these predictions. Furthermore, in the former age group, 3/9 and in the latter, 3/6 were less experienced with modeling topics.

As discussed, and agreed upon by the interviewer and interview partners, there was a trade-off in HPV spread modeling between a simple model with a small confidence interval of its predictions, which models only a small part of reality with greater precision, and a sophisticated model with a larger confidence interval of its predictions, which models a more realistic environment with less precision. A conversation about simple and sophisticated mathematical models helped to contextualize their usages (Supplementary Table 3, excerpt 2).

Both interviewer and interviewee preferred a simple model but agreed that the Ethiopian context had to be considered when choosing an appropriate model and that users of a model must acknowledge the trade-off between a simple and a sophisticated model.

Most participants said that they preferred a simple model over a sophisticated one, preferring less realistic but more precise predictions over high-resolution models. Some decision-makers would like to start with a simple model and move towards more sophisticated ones over time. Health decision-makers generally trusted mathematical models and having a team of specialists was viewed as helpful to solve the trust problem concerning models and computer simulations, as generated from this piece of conversation (Supplementary Table 3, excerpt 3).

One participant expressed the wish to understand the mathematical model more deeply to trust it (Supplementary Table 3, excerpt 4).

### Outlook and recommendations of decision-makers

Mathematical modeling and computer simulations were seen favorably by health decision-makers and were either being used by decision-makers, or they were very interested in adopting them in the future. They hoped predictions will help with various problems in the country and encourage to promote interventions. As interviewees suggested, models might aid in determining the incidence and prevalence of CC, as well as the most common serotypes. A decision-maker expressed optimism about the development of an HPV transmission model and emphasized the importance of the input data quality (Box 3, excerpt 1).

Digitization and computer modeling is perceived as a chance (Box 3, excerpt 2).

However, health decision-makers had a few recommendations for modelers working with mathematical models. Obtaining accurate baseline data and emphasizing high-quality input data was highlighted as most important stage for generating credible predictions. One participant just exclaimed: *“Data, reliable data!”(….)* when asked for recommendations.

Furthermore, because Ethiopia has many ethnic groups, each with their own culture, religion, and language, health decision-makers believed that taking cultural factors into account in the local Ethiopian setting is critical (Box 3, excerpt 3).

Participants emphasized the necessity of re-evaluating and discussing a mathematical model after introducing it and underscored the need for constant improvement (Box 3, excerpt 4). Specific recommendations were: consider immune profiles; model investment return; talk to the fieldworkers; try to promote the importance of modeling; use software which is freely available in Ethiopia and present the model in simplified ways.

Box 3 Outlook and recommendations of decision-makers **Excerpts from interviews:**

1. **IP:** I am much more hopeful on this one as this modeling is probably going to be done on the information you are going to gather in Ethiopia. (MOH, health decision-maker)
2. **IP:** Because data is freely available and it can level the field, I mean between the north and the south. And fortunately, the young generation is fast grasping up the technique and so on. So, I mean this is an opportunity that you don’t miss. I mean, with the age of digitization and so on, this is a big opportunity for Sub-Saharan Africa. Not only to catch up, even in some areas to excel… (PHS, advisor)
3. **IP:** …because we have a lot of conservative societies in Ethiopia in some areas. Because of religion, cultural, social … constructs are different. So, I think this needs to be properly understood for effective intervention using HPV vaccination in Ethiopia. That has not been done like, it’s very important… (MOH, health decision-maker)
4. **IP:** I don’t think that the reasons that you get from modeling is something that remain a reality forever. It is something that is dynamic, and you are going to review and change it at some point and um… you are learning, you are learning [and] exercise will also going to help you improve your model. I think we need to leave that kind of room for your modeling. So that this modeling is open for, you know, depending on your experience in the future. (MOH, health decision-maker)

## Discussion

Our qualitative study used deliberative interviews in Ethiopia to investigate the information needs of health decision-makers concerning mathematical modeling and computer simulations for sustainable decision-making and highlighted much interest and openness among health-decision makers towards the use of mathematical models and computer simulations. Most of the decision-makers interviewed consider the usage of theoretical predictions in their future decision making or are already using them. However, HPV data are still scarce and there is a need to allocate resources to high quality data generation, while considering the general resource constraints.

The involvement of experts working on mathematical models in the decision-making may contribute to decision-makers’ understanding and help to apply the mathematical predictions in the decision making. As our participants explained their most useful data sources are the DHIS2 and the DHS, which resonates with work from Ozodiegwu and colleagues who confirm the usefulness of Demographic and Health Surveys for data concerning Malaria to inform Malaria transmission models in Nigeria^30^.

Identifying hot spots for HPV infections was difficult for interviewees. Decision-makers tend to link HPV infections with HIV infections and assume that a higher prevalence of HPV occurs in locations with a high prevalence of HIV infections. This is consistent with research indicating that HIV infection promotes HPV infection and vice versa^31–33^. Hence, there might be a higher prevalence in urban areas with a higher prevalence of HIV infections and a higher number of unsafe sexual encounters. This has been seen in work by Maulide Cane and colleagues, who demonstrate that with the verification of higher HIV prevalence in urban areas among young women and men than in rural areas in sub-Saharan Africa^34^.

Decision-makers showed particular interest regarding information on the national and local burden of HPV; vaccination thresholds for herd immunity and the reproductive number R; cost-effectiveness and acceptability of health interventions due to cultural aspects. Calculating vaccination thresholds were also a critical parameter in an HPV transmission model proposed by Riesen and colleagues^13^.

Cultural elements specific to Ethiopia were also underscored as very important. As our participants recommended, when modeling HPV prevalence, these cultural factors should be included in the model. Sociocultural barriers to vaccination and other means of CC prevention were also seen to play a crucial role in Ethiopia. Brandt and colleagues highlighted these sociocultural barriers in a study of the acceptability of HPV self-collection in Ethiopia^35^ which emphasizes that the different cultural aspects of more than 80 different ethnic groups^36^ should be further discussed between modelers and decision-makers in the process of model creation.

Vaccination for boys and girls, versus female-only, can be resource efficient. Only 5% of low-income countries provided vaccination for males, compared to over 44% of high-income nations, in 2019^22^. Paucity of evidence for cost-effectiveness in LMICs^37^, and particularly worldwide HPV vaccine shortage^22,38^ hampered the introduction of HPV vaccinations for boys in LMICs like Ethiopia. In a modeled scenario where vaccinating boys (aged 9–14) or women aged ≥ 18 was considered, Drolet and colleagues found less cost-effectiveness^22^. Ethiopia started an HPV-vaccination program for girls aged 14 in late 2018 with Gardasil-4TM (HPV 6, 11, 16, 18)^39^. A systematic review of 10 Ethiopian studies, including 3633 women with different kinds of cervical abnormalities, identified HPV 16 (37.3%), 52 (6.8%), 35(4.8%), 18(4.4%)) to be the most prevalent genotypes^39^, which calls for vaccines with a broader scope as HPV 18 might be of lesser importance.

We used deliberative interviews as proposed by Berner-Rodoreda and colleagues^27^ because we wanted to co-create knowledge by mutually drawing on each other’s expertise; jointly deliberate to add different facets to complex problems; share personal experiences to generate more nuanced insights; and build a friendly conversational environment leading to additional volunteered, even more personal, information. Our analysis showed positive rapport in the majority of the interviews, and it seems that it was easier to create good rapport with individuals of similar age and educational level. Age and personality of the interviewees appears to have had a stronger influence on rapport than their professional position.

Berner-Rodoreda and colleagues compared deliberative interviews and conventional qualitative interviews with health experts in Germany^40^. We also observed that personality, professional position, age, education and background knowledge on discussed topics seemed to influence speaking time and deliberation^40^. The high-ranking officials and talkative personalities spoke more and with greater freedom, but we did not observe a significant difference in female and male interview partners in this regard.

We found strengths and limitations in this new interview-style. Our experiences with the deliberative interview style were similar to those described by Berner-Rodoreda and colleagues: the created environment and the conversational interview resulted in a relaxed atmosphere, the generation of more detailed information, and, in some circumstances, the production of enhanced joint knowledge^40^. The sharing of the interviewer’s experiences from a local hospital, led to more significant information volunteered by the interviewee. Furthermore, new themes evolved, such as cultural elements regarding the vaccination for girls. On the other hand, we observed some difficulties. Even though we briefed interview partners thoroughly beforehand, it was challenging to establish an equilibrium of knowledge in some cases, particularly concerning mathematical models. In the future, a more selective approach in choosing interview partners might be advantageous. Moreover, the positionality of the interviewer and interviewee may make it difficult to have a mutually challenging dialogue.

In terms of roles, we also noted that an exchange of role of interview partner and the interviewer was rare due to the interviewee’s habits being used to the conventional interview-styles^40^. In a few cases, we also encountered a change of mind in the interviewee, though this was rare because the interviewer and interview partners agreed on most topics. We agree with Berner-Rodoreda and colleagues on the importance of pre-briefing the interviewees to improve the quality of the deliberative interview^40^ as in our case, the briefing allowed participants to reflect on the mathematical models and simulations previously discussed and contributed to an eye-to-eye conversation about these topics that may have helped create further insights. We found that not all research topics might be equally amenable to the use of deliberative interviews. If the specific interview topic allowed for new ideas or collaboration to innovate, it was easier to deliberate and co-generate knowledge. Facts about the HPV burden in Ethiopia did not leave as much room to deliberate as the future of mathematical modeling in Ethiopia or screening and vaccination. We suggest examining the potential outcome of the interviews closely before choosing the deliberative interview. IPs holding monologues or veering off the topic could be attributed more to personality and work experience, rather than age and gender, in our study^40^.

Overall deliberative interviews proved to be useful in interviewing Ethiopian health decision-makers. There are, however, some limitations in our study. The recruitment of health decision-makers was restricted to Addis Ababa and Gondar only. Asking health decision-makers from other regions might have brought out additional facets which may be more important in other parts of the country and although we interviewed key-people we could not cover all cultural aspects of the vastly diverse Ethiopian society. Further studies on the different cultural aspects are warranted. Another limitation was the gender composition of our sample with only one female participant. A second potential female candidate refused participation for unknown reasons and recommended a male participant. This may be because women have not traditionally held positions of decision-making and are still underrepresented in higher education, leadership positions, and decision-making^41–43^.

When Opoku and colleagues learned about the perceptions of Ghanaian policymakers on mHealth interventions^23^ their interviews lasted 45 min on average. The duration is comparable to our interviews. Besides, Yesuf and colleagues used in-depth interviews and focus-group discussions to learn from Ethiopian administrators and health care providers, which was also different from our approach^24^. While Opoku and colleagues and Yesuf and colleagues used conventional qualitative interviews and focus-group discussions to gain an understanding of mHealth interventions and healthcare systems, our approach of using deliberation with interview partners yielded more information on information needs for Ethiopian policymakers but may have been a drawback in terms of evoking data from participants with less knowledge about mathematical models.

As computer science and data-driven prediction models advance, Ethiopian health decision-makers are likely to make more and more use of them. Since decision-makers prefer mathematical models based on the Ethiopian setting and local data, there will be a great need for establishing models tailored to Ethiopia. The results of this study may help to find appropriate models to tackle public health problems like CC deaths in Ethiopia and tailored simulations of HPV spread might help in finding appropriate screening strategies to reduce the burden of disease. Furthermore, we underline the necessity of the preceding interview briefing in order to optimize the success of the deliberative interview. For deliberative interviews including issues needing in-depth expertise, a knowledge-based pre-selection of the interview partner may be useful. Further research needs to be conducted on the compatibility of deliberative interviews with different research topics, alongside more studies on information needs, specifically cost information, for health decision-makers for improved health decision-making in African countries.

## Methods

### Setting

This research was conducted in Addis Ababa and Gondar, Ethiopia from October 29^th^, 2019 to November 11^th^, 2019. We chose these cities since they locate many health decision-makers.

### Ethical approval

The institutional review boards of the University of Addis Ababa and Heidelberg University approved this study. All participants provided written informed consent before the interviews were conducted. Every action taken by the authors was in accordance with the Declaration of Helsinki and the Global Code of Conduct. Local researchers were included throughout the research process to ensure the research is locally relevant.

### Sampling and data collection

Purposive sampling was used to recruit participants from Ethiopia. Participants were eligible for inclusion if they were directly involved in health decision-making or had an influential role on decision-makers in the field of HPV and cervical cancer prevention either at national or local level. They were initially approached via E-mail. We contacted 23 people, 15 of whom agreed to participate in the study. No response (*n* = 4), not being eligible for the study (*n* = 2), or refusal and appointment of another individual (*n* = 2) were the reasons for non-participation.

We interviewed health decision-makers with a novel interview technique, the ‘deliberative interview’, which is described as debate-oriented and as aiming to achieve equality in the interviewer-interviewee relationship^27^. Pre-briefing of participants is also an integral part of the deliberative interview, which potentially generates more knowledge than existing interview styles^27^.

Before collecting data, researchers and interview partners were thoroughly pre-instructed on the novel interview-style. Prior to the interview, participants received an information sheet with basic background information on the models to be addressed and instructions on how to conduct a deliberative interview (Supplementary Methods). Additionally, we verbally pre-briefed interviewees on the models and the interview format before recording the interview. Before the study, there was no relationship between interviewers and participants. We developed an interview guide of 16 open-ended questions and suggestions for conducting a debate- or conversation-oriented interview, see Supplementary Methods. Participants could choose a convenient place and time for the interviews, which were mainly conducted in participants’ offices or at a quiet place to avoid disturbances. Only participants and interviewers were present. Interviews were conducted in English by FS and HA, lasted between 25 and 60 min and notes were taken during the interview. The interviews were recorded and verbatim transcribed. Data collection reached saturation when no new topics emerged from the interviews^44^.

### Analysis

Data analysis was conducted in line with the five steps of the framework approach: familiarization, identifying a thematic framework, indexing, charting, mapping and interpretation^45^. We drew on thematic analysis^46^. After familiarization with the primary data, we developed a codebook by deductively and inductively generating codes in an iterative process using the software NVivo 12 Pro. Codes were agreed upon by the research team. FS coded all interviews; two randomly selected interviews were co-coded by ABR. Both coders showed the same understanding and application of the codes thus building inter-coder reliability. Minor discrepancies related to the code “expert/non-expert” which had been described ambiguously. The discrepancy of who it should apply to was resolved after discussion. The overall themes identified from the interviews were data reliability and resource constraints with pertinent sub-themes presented in the results section.

### Reporting summary

Further information on research design is available in the Nature Research Reporting Summary linked to this article.

## Supplementary information

Supplementary Information

REPORTING SUMMARY

## Data Availability

The full transcripts of the interviews and audio files are not available to the public due to data protection reasons. Metadata, like the code tree, that support the findings are available from the corresponding author upon reasonable request.

## References

[1] Sung H (2021). Global cancer statistics 2020: GLOBOCAN estimates of incidence and mortality worldwide for 36 cancers in 185 countries. CA: Cancer J. Clin..

[2] IARC. Cervix cancer screening. IARC handbooks of cancer prevention, International Agency for Research on Cancer. *World Health Organization*. *IARC Press* (2005).

[3] Ferlay, J. et al. *Global Cancer Observatory: Cancer Today*. *Lyon, France*: *International Agency for Research on Cancer*, <https://gco.iarc.fr/today> (2020).

[4] Schiffman M, Castle PE, Jeronimo J, Rodriguez AC, Wacholder S (2007). Human papillomavirus and cervical cancer. Lancet.

[5] Walboomers JMM (1999). Human papillomavirus is a necessary cause of invasive cervical cancer worldwide. J. Pathol..

[6] WHO. *Global Health Expenditure Database*, <https://apps.who.int/nha/database/Select/Indicators/en> (2022).

[7] WHO guideline for screening and treatment of cervical pre-cancer lesions for cervical cancer prevention, second edition. Geneva: World Health Organization; 2021. Licence: CC BY-NC-SA 3.0 IGO. 34314129

[8] Hall MT (2020). The past, present and future impact of HIV prevention and control on HPV and cervical disease in Tanzania: a modelling study. PloS one.

[9] Hontelez JA (2021). Evidence-based policymaking when evidence is incomplete: The case of HIV programme integration. PLoS Med..

[10] Nyabadza F, Mukandavire Z, Hove-Musekwa SD (2011). Modelling the HIV/AIDS epidemic trends in South Africa: Insights from a simple mathematical model. Nonlinear Anal.: Real. World Appl..

[11] Dehning, J. et al. Inferring change points in the spread of COVID-19 reveals the effectiveness of interventions. *Science* **369**, 10.1126/science.abb9789 (2020). 10.1126/science.abb9789 PMC7239331 32414780

[12] Ferrari A (2021). Simulating SARS-CoV-2 epidemics by region-specific variables and modeling contact tracing app containment. NPJ Digit. Med..

[13] Riesen M, Garcia V, Low N, Althaus CL (2017). Modeling the consequences of regional heterogeneity in human papillomavirus (HPV) vaccination uptake on transmission in Switzerland. Vaccine.

[14] Goldie SJ (2005). Cost-effectiveness of cervical-cancer screening in five developing countries. N. Engl. J. Med..

[15] Goldie SJ, Kuhn L, Denny L, Pollack A, Wright TC (2001). Policy analysis of cervical cancer screening strategies in low-resource settingsclinical benefits and cost-effectiveness. JAMA.

[16] Tracy L (2011). Estimating the impact of human papillomavirus (HPV) vaccination on HPV prevalence and cervical cancer incidence in Mali. Clin. Infect. Dis.: Off. Publ. Infect. Dis. Soc. Am..

[17] Hughes JP, Garnett GP, Koutsky L (2002). The theoretical population-level impact of a prophylactic human papilloma virus vaccine. Epidemiol. (Camb., Mass.).

[18] Bearman PS, Moody J, Stovel K (2004). Chains of affection: The structure of adolescent romantic and sexual networks. Am. J. Sociol..

[19] Liu G (2022). Impact of catch-up human papillomavirus vaccination on cervical cancer incidence in Kenya: A mathematical modeling evaluation of HPV vaccination strategies in the context of moderate HIV prevalence. E. Clin. Med..

[20] van Schalkwyk C, Moodley J, Welte A, Johnson LF (2021). Modelling the impact of prevention strategies on cervical cancer incidence in South Africa. Int. J. Cancer.

[21] Tan N (2018). Model-estimated effectiveness of single dose 9-valent HPV vaccination for HIV-positive and HIV-negative females in South Africa. Vaccine.

[22] Drolet M (2021). Optimal human papillomavirus vaccination strategies to prevent cervical cancer in low-income and middle-income countries in the context of limited resources: A mathematical modelling analysis. Lancet Infect. Dis..

[23] Opoku D, Busse R, Quentin W (2019). Achieving Sustainability and Scale-Up of Mobile Health Noncommunicable Disease Interventions in Sub-Saharan Africa: Views of Policy Makers in Ghana. JMIR Mhealth Uhealth.

[24] Yesuf EA, Grill E, Fröschl G, Koller D, Haile-Mariam D (2019). Administrators, health service providers, and consumers perspectives of functions of district health-care systems in Oromia region, Ethiopia: A qualitative study. Int. J. Health Plan. Manag..

[25] Brinkmann S (2007). Could Interviews Be Epistemic?: An Alternative to Qualitative Opinion Polling. Qual. Inq..

[26] Rogers CR (1945). The Nondirective method as a technique for social research. Am. J. Sociol..

[27] Berner-Rodoreda A (2020). From doxastic to epistemic: A typology and critique of qualitative interview styles. Qual. Inq..

[28] CSAAA. *Demographic and Health Survey 2016*, <https://dhsprogram.com/pubs/pdf/FR328/FR328.pdf> (2017).

[29] Ali KE (2019). Burden and genotype distribution of high-risk Human Papillomavirus infection and cervical cytology abnormalities at selected obstetrics and gynecology clinics of Addis Ababa, Ethiopia. BMC Cancer.

[30] Ozodiegwu ID (2021). Beyond national indicators: Adapting the Demographic and Health Surveys’ sampling strategies and questions to better inform subnational malaria intervention policy. Malar. J..

[31] Palefsky J (2006). Biology of HPV in HIV Infection. Adv. Dent. Res..

[32] Zayats R, Murooka TT, McKinnon LR (2022). HPV and the risk of HIV acquisition in women. Front Cell Infect. Microbiol.

[33] Stelzle D (2021). Estimates of the global burden of cervical cancer associated with HIV. Lancet Glob. Health.

[34] Maulide Cane R (2021). HIV trends and disparities by gender and urban–rural residence among adolescents in sub-Saharan Africa. Reprod. Health.

[35] Brandt T (2019). Genital self-sampling for HPV-based cervical cancer screening: A qualitative study of preferences and barriers in rural Ethiopia. BMC Public Health.

[36] Van der Beken C (2007). Ethiopia: Constitutional protection of Ethnic minorities at the regional level. Afr. Focus.

[37] Ng SS, Hutubessy R, Chaiyakunapruk N (2018). Systematic review of cost-effectiveness studies of human papillomavirus (HPV) vaccination: 9-Valent vaccine, gender-neutral and multiple age cohort vaccination. Vaccine.

[38] World Health Organization = Organisation mondiale de la Santé. (2019). Weekly Epidemiological Record, 2019, vol. 94, 47 [full issue]. Weekly Epidemiological Record = Relevé épidémiologique hebdomadaire, 94, (541– 560. World Health Organization = Organisation mondiale de la Santé. https://apps.who.int/iris/handle/10665/329962

[39] Derbie A (2022). Human papillomavirus genotype distribution in Ethiopia: An updated systematic review. Virol. J..

[40] Berner-Rodoreda A (2021). “Thought provoking”,“interactive”, and “more like a peer talk”: Testing the deliberative interview style in Germany. SSM-Qual. Res. Health.

[41] Molla T, Cuthbert D (2014). Qualitative inequality: Experiences of women in Ethiopian higher education. Gend. Educ..

[42] Kassa S (2015). Challenges and opportunities of women political participation in Ethiopia. J. Glob. Econ..

[43] Alemayehu E (2021). Gender and leadership in Ethiopian higher education: Challenges and opportunities: The case of Addis Ababa University. Acad. Educ. Leadersh. J..

[44] Morse JM (2000). Determining sample size. Qual. Health Res..

[45] Pope C, Ziebland S, Mays N (2000). Qualitative research in health care. Analysing qualitative data. BMJ.

[46] Braun V, Clarke V (2006). Using thematic analysis in psychology. Qual. Res. Psychol..

