# Supplementary material for: Exploring computer-aided health decision-making on cervical cancer interventions through deliberative interviews in Ethiopia
Source: NPJ Digit Med. 2023 Apr 17;6:68. doi: 10.1038/s41746-023-00808-9 (PMC10106317; doi:10.1038/s41746-023-00808-9)
Supplement: Supplementary file 1 — Supplementary Information [file 41746_2023_808_MOESM1_ESM.pdf]

Supplementary Methods:

- Interview guide
- Information sheet

Supplementary Tables:

- **Supplementary Table 1:** HPV and cervical cancer burden in Ethiopia
- **Supplementary Table 2:** HPV screening and vaccination
- **Supplementary Table 3:** Significance of mathematical modeling and computer simulations

Interview guide

(This is a preliminary guide and will be adapted according to qualitative research)

I would like to thank you for taking the time to meet with me today. My name is Frithjof Sy from the HIGH in Heidelberg (Germany) and I would like to talk to you about your experiences as a health policymaker.

We are interested in talking with you about information you need in order to conduct good health decision making concerning the HPV burden in Ethiopia. Human papillomavirus infections are the foremost reason for developing invasive cervical cancer (ICC), particularly in Zambia and Malawi. Ethiopia takes its share equally.
We collect data and use mathematical equations to predict vaccination coverage or screening thresholds and are interested in talking with you to get a more detailed picture and improve our model and predictions.
The interview type, we are going to conduct is a so-called deliberative interview. The difference to a conventional interview is that the deliberative interview rather is a dialogue between you and me. It is a two-way conversation, where I encourage you to ask questions based on the background information we provided you with or questions which will arise during our conversation.

Consent and recording

Before we start the interview may I kindly ask you to read and, if you agree, sign the consent form? Your personal data will not be used in any publication. The participation is voluntary and you may withdraw from the study at any time.

The interview should take about 60 minutes. I will be taping the session because I don’t want to miss any of your comments. **All responses will be kept confidential**. This means that your interview responses will only be shared with research team members and we will ensure that any information we include in our report does not identify you as the respondent.

To begin with, may we fill in your personal data together? As mentioned, your personal data will only be accessible by the research team.

Deliberative Interview (DI)<br>(to be filled in by the interviewer)	
Name of the file:<br><br>**Institution (MED, NGO, POL, MOH, PHS)/ initials of interviewer (FS)/ # of interview of the day/ date (day month year)**<br>**e.g. MED.FS.1.29.04.2019**<br>**(MED-medical doctor, NGO-member of non-governmental organization, POL-policymaker, MOH-member of ministry of health, PHS-public health specialist)**	
Interview Date (Day-Month-Year)	
City	
Institution/Professional Group	
Interviewer	
Transcriber	
Translator	
Duration of interview (in minutes)	

Information about Interviewee<br>(to be filled in before or at the end of the interview)	
Age	
Gender	
Present Position	
Educational level (name highest degree or educational level)	
Profession	
How long have you worked in the field of health?	

Are there any questions from your side before we start?

Thank you very much Mr. _________________for a agreeing to be interviewed. We will be talking about your information needs for being able to make good health decisions on interventions in the field of HPV and cervical cancer. We will conduct a deliberative interview, which will be at some points a two-way conversation, in our case. To begin with…

Questions:

1. … To begin with can you describe your involvement in health decision making?

    ***Personal notes**: We are interested in the topic of HPV. Can you tell me what data resources concerning HPV…*

2. …Can you tell me what data resources concerning HPV you use in your work?
    - Do other data sources exist?
    - What are the reasons why you do not use those?
    - Which ones are most useful to you?
        - Why?

    ***Personal notes**: Why do you not think that …. (vaccination thresholds and screening thresholds) would be useful? In my opinion… (it is useful because we can make future predictions) What do you think about that?*

3. From your experience, which geographic area in Ethiopia has the highest HPV burden?
4. Is HPV vaccination and screening a priority for you?
    - If yes, why
    - If not, why not?

    ***Personal notes:** counter arguments: if not: - vaccination will elude future cervical cancer occurrence. Screening will reduce cervical cancer burden of not yet vaccinated women. If yes: -e.g. aren't diarrheal diseases more important to consider because they affect more people?*

5. How high is the burden of cervical cancer in Ethiopia? Can you describe please.
6. How would you describe the connection between HPV infections and the development of cervical cancer in Ethiopian women?
    - From your experience, which are the main triggers of cervical cancer?

***Personal notes:** In many cases a clear connection can be drawn. Scientific studies found a close to 100% involvement of HPV in the studied cervical cancer samples.*

7. How do you view the issue of resources in the context of health interventions in Ethiopia?

   ***Personal notes**: I agree/disagree. To my knowledge, there is a lack of resources in Ethiopia concerning means to tackle the HPV burden. By far not every community can be offered a vaccination or screening health intervention.*

8. Which information do you regard as necessary to make decisions about resource allocation?

   ***Personal notes**: I also think that.... (information about vaccination thresholds and screening thresholds are important, because it might help to identify delicate communities) It would be crucial… (to know about..) What do you think about that?*

9. What do you need to know about local HPV vaccination and screening in order to make a good decision concerning health interventions? Please elaborate
   - Which information do you consider most important?

   ***Personal notes**: In my opinion information about vaccination and screening thresholds in different communities could be useful, as well as information about the seat of infection (spatial). What is your opinion about that? Are there other aspects that are important to you? For me ..... is also important.*

   ***Personal notes**: Mathematically informed decision-making can improve conventional decision-making. Within this approach mathematical equations can describe Human papillomavirus spread based on real-life data taken from populations under investigation. On simulating different scenarios, the best scenario could guide the decision-making.*

10. What is your view on mathematical informed decision-making?
    - How useful do you think this might be for your work?

    ***Personal notes**: It is already widely used to predict future health scenarios. Not only in population dynamics, as well in cancer therapy*

mathematical informed computer simulations predicted future therapy options.

11. How useful do you regard computer simulations?

    Personal notes*: In my view, computer simulations (CS) will more and more redefine our societies. CS already delivered predicted information for policymakers in Switzerland e.g. (Riesen et. al.)*

12. How useful do you think predictions based on mathematical models are?

    Personal notes*: It is already scientifically introduced and used for future predictions. A SVIR-ODE-Model was used by Riesen et. al.*

    Personal notes: *Only mention this question if participant is familiar with mathematical modelling*

13. What are your hopes and expectations towards theoretical predicted results?
14. What recommendations do you have for modellers like me?
15. If you had the choice between a simple and a sophisticated prediction tool which one would you prefer?
    - Can you give reasons why?

    Personal notes: *My opinion on sophisticated tools: Computer algorithms can process more data at once compared with our human brain. With this in mind the computer can consider more information simultaneously, which might guide us as humans.*

16. Is there anything else you would have liked to say on this topic that we have not talked about in our discussion?

Thank you very much for your time.

<table>
<tr><th colspan="2">Comments and notes of the interviewer
(to be filled in by the interviewer after the interview)</th></tr>
<tr><td colspan="2">How well did this interview go?

What did work, what did not?

What may have to be done differently in the future?

Further notes:</td></tr>
<tr><td>Was the interview interrupted?

Tick: [ ] yes [ ] no</td><td>If yes, who interrupted?

interview was interrupted: [Insert number]:</td></tr>
</table>

Information Sheet
Frithjof Sy
Version 2.0
29.10.2019

Universitätsklinikum Heidelberg | Im Neuenheimer Feld 324 | 69120 Heidelberg

Information sheet

Title of the Study: Deliberative interviews of policymakers in Ethiopia asking information needs for improved health decision-making.

Dear participants,

Thank you very much for considering taking part in this study. Please take your time to read the following information carefully and do not hesitate to ask me in case you have questions.

My Name is Frithjof Sy, I am a PhD student of the Heidelberg Institute of Global Health (HIGH) affiliated with the University of Heidelberg in Germany. Under the supervision of Dr. Andreas Deckert and Prof. Dr. Dr. Till Bärnighausen in collaboration with Dr. Hermann Bussmann I am conducting this research study as part of my PhD-program. This study has been approved by the ethics committee of the Medical Faculty of Heidelberg and the Addis Ababa University Institutional Review Board(AAU-IRB). AAU CHS IRB reviewed and approved the informed consent as the other IRB counterparts did.

Aim of the study
Our objectives are to learn about the information needs of policymakers in Ethiopia in order for them to provide improved health decision-making, as a primary goal. Furthermore, we like to explore expectations of policymakers towards theoretical modelling of HPV spread. Identifying their views, hopes and concerns on theoretical modelling are of great importance to gain alignment of theory and reality. In detail, information needs about HPV vaccination and HPV screening shall be considered. You are asked to participate in this study because you might have particular experience or knowledge in health decision-making of HPV or cervical cancer interventions.

Background
Human papillomavirus infections are the foremost reason for developing invasive cervical cancer(ICC). Sub-Sahara Africa bears the big part of this burden. Countries like Zambia and Malawi have the highest prevalence of ICC age-standardized rates(ASR) worldwide. Ethiopia takes its share equally, here the ASR of cervical cancer represents 18.9 per 100.000 women, which is almost triple the ASR of the USA with 6.5.5/100.000. Our group "Epidemiology and Biostatistics" associated with the Heidelberg Institute of Global Health(HIGH) in Heidelberg (Germany) wants to use mathematical modelling of HPV infections built on well-established models widely utilized to help generate this information with the simulation of HPV spread in silico (within a computer simulation). Mathematical models can make predictions to guide health decision making. Questions

like: In which risk areas are vaccination urgent? Which age groups should be screened for HPV infection? Should risk populations be screened?

Study procedure

We like to conduct a qualitative study with deliberative interviews in a way proposed by Berner-Rodoreda et al. of up to 25 policymakers in Ethiopia. The deliberative interview is rather a conversation than an interview. Meaning not only me, but also you are very welcome to ask questions. On certain questions I will offer you an opinion as well as explanations if needed. The timeframe of the interview will be about 60 minutes.
The deliberative interview will be recorded with a voice recorder. The voice-recording will be transcribed followed by extracting information. The results of the investigation will be published and will influence our theoretical model of HPV spread in Ethiopia. Data-protection will be guaranteed, it will be possible to join the data with your name by the participated researchers.

Personal risk

The researcher understands that some topics, especially talking about genital HPV sample taking may be a sensitive topic to some participants. Participants are encouraged only to talk about topics they are willing to share.
Benefits: There are no direct personal benefits for participating in the study. However, as a participant of this study, your thoughts and suggestions are important to align the theory of mathematical modelling and reality. As your expertise gives opportunities to inform mathematical models to improve future health decision-making.

Voluntary participation and right to withdraw

Your participation in the study is voluntary. You may pause or withdraw from the study at any time. If you do not want to answer any specific question you are encouraged to speak up and we will move on to the next question. Refusal of participation in the study or withdrawal from it will bear no consequences for you. In case of withdrawal from the study all your collected data will be deleted if you wish so. If you change your mind about participation you can contact me or another member of our team.

Data protection

This study complies with the data protection law of Ethiopia. During the study we will take personal data from you. The data will be kept with the study coordinator and will be stored electronically. Data important for the study will additionally be stored in pseudonymised[1] form and used by the research for analysis. However, data will not be passed on to third parties outside the University of Heidelberg in Germany. The study management will take all appropriate steps in order to guarantee the protection of your data according to the laws in Ethiopia and the data protection standards of the European Union. Data are protected against unauthorized access. Decryption will be only done if the study reveals a health risk of a participant which requires immediate medical action. Data will be utilized exclusively for the purpose of this study. You have the right to request information from the responsible person (see below) about all your stored personalized data. You can also request the correction of incorrect data, as well as the

[1] "Pseudonymisation" means processing of data in such a manner that personalized data can't be allocated to a specific person without enlistment of additional information ("keys"). The additional information will be kept separately and are subject to technical and organizational measures which guarantee that the personalized data cannot be allocated to an identified or identifiable person.

deletion of data or the limitation of their processing. After data analysis, all data will be deleted. Voice recordings will be deleted after 2 years, transcriptions will be deleted after 5 years. In case of improper data processing you have the right to complain to a supervisory board authority.

Person in charge of Data processing of the study data:
Frithjof Sy
Bergheimer Straße 137
69115 Heidelberg
Germany
Phone: +4917656206706
Email: F.sy@stud.uni-heidelberg.de

Data protection officer of the University of Heidelberg:
Dr. iur. Regina Mathes
Grabengasse 1
69117 Heidelberg
Email: datenschutz@uni-heidelberg.de

publication:

After finishing the study I might publish the study process and the results. The main findings will be presented anonymously. Your personal data will not appear in any part of the study.

If you have any further questions, please do not hesitate to ask me in person during the interview or contact me (see information below) at any time.
Thank you very much for participating in our study

Frithjof Sy
Email: F.sy@stud.uni-heidelberg.de

Contact address of CHS IRB:
Phone: +251118961396
Email: chs.irb@aau.edu.net

Supplementary Table 1: HPV and cervical cancer burden in Ethiopia

Excerpts from interviews:

1. **IP**: …in the rural areas, it's very difficult for a woman, because women are not really decision-makers in the family, in the rural areas. If you want... if she wants to seek healthcare, she has to get the permission from her husband and that would take probably hours of travel to get there... and they might think that they have to pay for the service, even though, they don't need to pay for the service, they might think that they have to pay for the service. And women are economically dependent on men in rural Ethiopia. (MED, advisor and decision-maker)
2. **IP**: …HPV is a sexually transmitted disease... so, in areas where there is urbanization, there is high prostitut[ion] and there is high sexual activity... it is possible, but I don't have a firm justification to say so. So definitely HPV will be very high in areas where there is high sexuality. (MOH, decision-maker)
3. **IP:** you see, the HPV burden is not... um... very well studied. It is in a few studies, like in Gondar, there was a study that says there was a high prevalence.
   I: yes.
   IP: it says the incidence is so high... I think especially... we can extrapolate from HIV prevalence because both of them are sexually transmitted, so I think mostly the metropolitan cities, the major cities are highly prevalent, but it needs study [for Ethiopia]. (MOH, decision-maker)
4. **IP:** Compare with the lowland areas. Most of this population and near to border areas. They have the highest HIV incidence.
   I: So, near to the Sudan border, you mean? Or which?
   IP: All the Ethiopian borders. In the trade centers, they have the highest incidence of HIV this will lead us… they might have the highest cervical cancer. For example, in Gondar. By observation, we can say that cervical cancer is more common in HIV-positive individuals.
   I: Ok, do you know why there are more HIV-infected and so probably HPV infected people at the border and lowlands. Do you have any explanation for that?
   IP: Boarder areas, you know, there might be long truck drivers..
   I: Ah, truck drivers?
   IP: Long truck drivers from the Ethiopian boarders also... and based on that they, the long truck drivers will stay for [a] long period of time with... without seeing their families and their wives, so they have a long period of time without their wives, and they might practice unsafe sexual activity. Um… and there may be also commercial sex workers. Um, they are doing business. Maybe that's the cause.
   I: Interesting, so the truck drivers are contributing to spreading the viruses HIV and HPV.
   IP: yeah, maybe they are. (PHS, health decision-maker)

Supplementary Table 2: HPV screening and vaccination

Excerpts from interviews:

1. **IP**: The second [most] common [genotype] from the data is not 18. You know it is other type, but also 18 is there. So, it could be some difference across populations, but since we don't have this concrete good data here, we generally need to use data from international sources. (MOH, health decision-maker)
2. **IP**: …the most critical ones are not only 16 and 18, but other HPV genotypes: 35, 45, 31, 33 which may not potentially cover with the current bivalent or quadrivalent doses. (POL, advisor and health decision-maker)
3. **I:** The Globocan states 16 and 18 would be the most prevalent in Ethiopia.
   IP: I think 18 there were recent studies done in Addis Ababa by EPHI, and they found either 16 or 18 are different. The current vaccine regiment doesn't cover all of the genotypes. That, even though the government has already brought out the quadrivalent vaccine, which covers 16, 18, 6 and 11. It doesn't cover all of the genotypes. So, it might not be effective. It might not be as effective as they think it is because there are other high-risk genotypes, like 31 and 33 and 35 and other high-risk ones.
   I: When I was talking to the other health decision-makers in Black Lions Hospital who are working as experts in the field. They told that 16 is maybe the most prevalent one, but 18 is not so much seen, actually..
   IP: Yes, that is it.
   I: So, the Globocan states something which is maybe not as accurate for Ethiopia in this case.
   IP: Yes...
   I: Which is quite interesting, I find. So, maybe more local studies would be necessary!
   IP: Sure, that's why we are here. Can your mathematical model take different types of genotypes into consideration? Um, some say two types of genotypes, or they say five types we don't know. They can't use maybe some of the types in this country.
   I: We were discussing this [in our team]. One option was just to model the HPV 16. Because this is the most prevalent one and of course we actually would have to know which genotypes are the most prevalent ones. It would definitely be of concern integrating more genotypes, but if we don't have data… Umm, it maybe does not make sense to use 31. So that's actually why we are thinking about just including 16, because for 16 everybody already was telling me this is the most prevalent one. I also thought about integrating 18, but now I learned it's actually not the most prevalent. So, I don't know, what do you suggest?
   IP: We are focusing on the most prevalent one. (PHS – advisor)
4. **I:** I encountered this actually when I was at Black Lions Hospital. I was walking in the isles with one of the interns and an elderly lady came up to us. Unfortunately, I could not understand, because she spoke in Amharic. One of the interns could translate and she was a patient with cervical cancer… …she had radiotherapy and she was waiting for follow-up.. which was maybe one year later and she was pleading, she was asking whether we could help her because she feared that she would die in between… …There are so many other patients who also have cervical cancer… yeah.. it was very sad seeing the lady and we tried going around and tried to talk to the doctors in the cancer department, but of course, it was not possible.
   IP: …that's why we tend to focus on prevention, screening and prevention. Because once the cases are advanced, it is very difficult to treat and also much more expensive to treat. So, we prefer to catch the woman at an early stage as possible. So that we can treat disease like with cryotherapy or other programs, other modalities of treatment. But once they reach the late stage.. it is extremely difficult and the outcome is not good. (MOH, health decision-maker)
5. **IP**: We need to map out who is… who has a stake in that girl, for the particular one girl here… …there are so many people behind her. From the peer-group, father, mother, grand-father, grand-father of mother's side, grand-father of father's side. They have different role[s].
   I: Yes, so but you would not say that it is like in most of the cases. And almost all cases the father. I mean, that's what I would expect.
   IP: You cannot conclude like that.

I: That would have been my first thought. Ok, the dad is going to make a decision and says… ok I like my girl to be vaccinated or not

IP: No, it is not like this. My point is. For the population of 100+ million in Ethiopia, you cannot have that kind of conclusion. Let me give you an example. If you go to, for example, in the very conservative Somali region of Ethiopia. Um.. or the Afar region with dominantly pastoralists. For example, when it comes to decision for children. There is a gender difference. If we are talking about the girl. The girl is expected to be like relatively less important to the boy, as he is going to get married and then leave[s] the family. So, the decision related to the girl might be left to the mother because it is less important. (MOH, health decision-maker)

6. **IP**: To do cytology you need trained cytologists in a country where you have maybe 200 pathologists for the whole nation and most located in the center of the country. It is not possible to have that screening methods. Once a woman develops cervical cancer the treatment, the number of … oncologists in the country is only 10… (MOH, health decision-maker)
7. **IP**: You know, you have to decide which one gets the machine and so on… So, resource limitation is really high. (MOH, health decision-maker)

Supplementary Table 3: Significance of mathematical modeling and computer simulations

Excerpts from interviews:

1. **I:** it is very useful. Um... I have only one critical, not critical… one very.. one issue that always concerns [me] when it comes to mathematical models... [A] mathematical model it gives you a scenery depending on the input you give into the model. (MOH, health decision-maker)
2. **IP**: The simple is preferred if I get all the output I want. But sometimes the simple prediction may not show the details.. so it depends whatever the case.. what I want is outputs, so that's what I want to say because I can't choose as a simple, easy prefer, complex prefer.. it depends on the data inputs and the expected outputs.
   I: We need the more complex ones! … so, it's important to consider the situation. What do we need and then if we need like more detailed information then it would be maybe important to, to fit it more to reality which means making it more sophisticated?
   IP: yeah
   I: In modelling, it is a trade-off. Between simple and sophisticated, sophisticated means more reality. Um.. but it also means, a wider confidence interval, so it is more unsure. So, the simpler it is the more sure the outputs are because you have more control over that what you put in, right?
   IP: Yeah, exactly! a trade-off! (MOH, health decision-maker)
3. **I**: What is your view on mathematical informed decision making? How useful do you think this might be for your work?
   IP: I think that is very important. We don't know exactly which is really the most significant contributor [for] the disease transmission dynamics. We don't have such information nowadays in this area. So, this is very important to have detailed mathematical modeling to come up with, [and ask] which factors are really potential contributing factors.
   I: What I can hold against it maybe is. Um, because sometimes mathematical models are complicated and um, it's difficult to grasp if you just look at it and if you do not have a background in, let say mathematics or in informatics, so sometimes it might be difficult to grasp how it works and then it's maybe a matter of trust. Wouldn't that be a problem?
   IP: It might not be a problem because you can just combine people while they are working on such issues for example there is a statistician. A statistician may not be perfect in mathematics but in the statistics and modeling designing they are perfect and there is [an] epidemiologist, there is a health informatician umm a bio-informatician. There is a basic science expert.
   I: This is actually a kind of network we are using.. so we are using a biologist, mathematicians maybe physicists, computer scientists, and they are working together as one big brain.
   IP: exactly! These things are the one! Yes!
   I: We can say. Um, if all these people are trustworthy and if they come together.
   IP: Yeah, exactly for a given project goal and aggregate for it. (EPHI, advisor and health decision-maker)
4. **IP**: …if I don't understand, it then would be vague and kind of scary. So, I think it is also the responsibility of people who design these to make sure that it is clear to me. So, I might not build the algorithm with you, but I need to understand what is it. (PHS, advisor and health decision-maker)
