# Supplementary material for: Exploring computer-aided health decision-making on cervical cancer interventions through deliberative interviews in Ethiopia
Source: NPJ Digit Med. 2023 Apr 17;6:68. doi: 10.1038/s41746-023-00808-9 (PMC10106317; doi:10.1038/s41746-023-00808-9)
Supplement: Supplementary file 2 — REPORTING SUMMARY [file 41746_2023_808_MOESM2_ESM.pdf]

nature portfolio

Corresponding author(s): Frithjof Sy, Andreas Deckert

Last updated by author(s): Mar 23, 2023

Reporting Summary

Nature Portfolio wishes to improve the reproducibility of the work that we publish. This form provides structure for consistency and transparency in reporting. For further information on Nature Portfolio policies, see our Editorial Policies and the Editorial Policy Checklist.

Statistics

For all statistical analyses, confirm that the following items are present in the figure legend, table legend, main text, or Methods section.

n/a	Confirmed	
☐	☒	The exact sample size (*n*) for each experimental group/condition, given as a discrete number and unit of measurement
☒	☐	A statement on whether measurements were taken from distinct samples or whether the same sample was measured repeatedly
☒	☐	The statistical test(s) used AND whether they are one- or two-sided<br>*Only common tests should be described solely by name; describe more complex techniques in the Methods section.*
☒	☐	A description of all covariates tested
☒	☐	A description of any assumptions or corrections, such as tests of normality and adjustment for multiple comparisons
☒	☐	A full description of the statistical parameters including central tendency (e.g. means) or other basic estimates (e.g. regression coefficient) AND variation (e.g. standard deviation) or associated estimates of uncertainty (e.g. confidence intervals)
☒	☐	For null hypothesis testing, the test statistic (e.g. *F*, *t*, *r*) with confidence intervals, effect sizes, degrees of freedom and *P* value noted<br>*Give P values as exact values whenever suitable.*
☒	☐	For Bayesian analysis, information on the choice of priors and Markov chain Monte Carlo settings
☒	☐	For hierarchical and complex designs, identification of the appropriate level for tests and full reporting of outcomes
☒	☐	Estimates of effect sizes (e.g. Cohen's *d*, Pearson's *r*), indicating how they were calculated

Our web collection on statistics for biologists contains articles on many of the points above.

Software and code

Policy information about availability of computer code

Data collection	no software was used for data collection
Data analysis	We used the software NVivo 12 Pro for data analysis.

For manuscripts utilizing custom algorithms or software that are central to the research but not yet described in published literature, software must be made available to editors and reviewers. We strongly encourage code deposition in a community repository (e.g. GitHub). See the Nature Portfolio guidelines for submitting code & software for further information.

Data

Policy information about availability of data

All manuscripts must include a data availability statement. This statement should provide the following information, where applicable:

- Accession codes, unique identifiers, or web links for publicly available datasets
- A description of any restrictions on data availability
- For clinical datasets or third party data, please ensure that the statement adheres to our policy

The full transcripts of the interviews and audio files are not available to the public due to data protection reasons. Metadata, like the code tree, that support the findings are available from the corresponding author upon reasonable request.

Human research participants

Policy information about studies involving human research participants and Sex and Gender in Research.

Reporting on sex and gender	The majority of the participants in our study (n=14) were men. Our study had just one female participant. This may be because women have not traditionally held positions of decision-making and are still underrepresented in higher education, leadership positions, and decision-making.
Population characteristics	See above
Recruitment	Purposive sampling was used to recruit participants from Ethiopia. Participants were initially approached via E-mail
Ethics oversight	The institutional review boards of the University of Addis Ababa and Heidelberg University approved this study.

Note that full information on the approval of the study protocol must also be provided in the manuscript.

Field-specific reporting

Please select the one below that is the best fit for your research. If you are not sure, read the appropriate sections before making your selection.

☐ Life sciences ☒ Behavioural & social sciences ☐ Ecological, evolutionary & environmental sciences

For a reference copy of the document with all sections, see nature.com/documents/nr-reporting-summary-flat.pdf

Behavioural & social sciences study design

All studies must disclose on these points even when the disclosure is negative.

Study description	This is a qualitative study of Ethiopian health decision-makers perceptions and information needs on data-driven computer-aided models concerning cervical cancer interventions. The research team conducted deliberative interviews, a novel interview style, with decision-makers in Addis Ababa and Gondar. The collected interviews were transcribed and coded in an iterative process drawing on thematic analysis.
Research sample	Study participants included 15 health decision-makers acting on national and local levels. They were working for the Federal Ministry of Health (FMOH) (n=5), the Ethiopian Public Health Institute (EPHI) (n=3), and other Ethiopian institutions like the Ethiopian Biotechnology Institute (EBTI) (n=1), Black Lions hospital (n=1), St. Paul's hospital (n=3), Family Guidance Association of Ethiopia (FGAE) (n=1); one was a public health officer (n=1). Participants were between 29 and 60 years old and mainly male (n=14) with one female participant.
Sampling strategy	The research team used purposive sampling to recruit participants from Ethiopia. They were eligible for inclusion if they were directly involved in health decision-making or had an influential role on decision-makers in the field of HPV and cervical cancer prevention either at a national or local level. The deliberative interviews were conducted until saturation was reached, such that no new information emerged.
Data collection	Researchers and interview partners were thoroughly briefed on the novel interview style before data collection. An interview guide of 16 open-ended questions and suggestions for conducting a debate- or conversation-oriented interview was developed by FS, ABR, AD, HB, and TB. Interviews were mainly conducted in participants' offices or in a quiet place to omit disturbances and recorded. The interviews were conducted by FS and HA in English. Only participants and interviewers were present. The interviews were transcribed and analyzed with Nvivo 12.
Timing	This research was conducted from October 29th, 2019 to November 11th, 2019 in Addis Ababa and Gondar, Ethiopia. It was con
Data exclusions	No data was excluded from the analysis.
Non-participation	Of 23 persons contacted eight did not participate for the following reasons. No response (n=4), not being eligible for the study (n=2), or refusal and appointment of another individual (n=2) were the reasons for non-participation.
Randomization	The participants were not allocated into experimental groups.

Reporting for specific materials, systems and methods

We require information from authors about some types of materials, experimental systems and methods used in many studies. Here, indicate whether each material, system or method listed is relevant to your study. If you are not sure if a list item applies to your research, read the appropriate section before selecting a response.

Materials & experimental systems

n/a	Involved in the study
☒	☐ Antibodies
☒	☐ Eukaryotic cell lines
☒	☐ Palaeontology and archaeology
☒	☐ Animals and other organisms
☒	☐ Clinical data
☒	☐ Dual use research of concern

Methods

n/a	Involved in the study
☒	☐ ChIP-seq
☒	☐ Flow cytometry
☒	☐ MRI-based neuroimaging
